# Advances in transcriptome analysis of human brain aging

**DOI:** 10.1038/s12276-020-00522-6

**Published:** 2020-11-26

**Authors:** Seokjin Ham, Seung-Jae V. Lee

**Affiliations:** Department of Biological Sciences, Korea Advanced Institute of Science and Technology, 291 Daehak-ro, Yuseong-gu, Daejeon, 34141 South Korea

**Keywords:** Ageing, Neural ageing

## Abstract

Aging is associated with gradual deterioration of physiological and biochemical functions, including cognitive decline. Transcriptome profiling of brain samples from individuals of varying ages has identified the whole-transcriptome changes that underlie age-associated cognitive declines. In this review, we discuss transcriptome-based research on human brain aging performed by using microarray and RNA sequencing analyses. Overall, decreased synaptic function and increased immune function are prevalent in most regions of the aged brain. Age-associated gene expression changes are also cell dependent and region dependent and are affected by genotype. In addition, the transcriptome changes that occur during brain aging include different splicing events, intersample heterogeneity, and altered levels of various types of noncoding RNAs. Establishing transcriptome-based hallmarks of human brain aging will improve the understanding of cognitive aging and neurodegenerative diseases and eventually lead to interventions that delay or prevent brain aging.

## Brain aging: RNA profiling to elucidate cognitive decline

Extensive exploration of RNA changes in the human brain over time should provide hallmarks for cognitive decline and neurodegenerative diseases. Changes in the messenger RNA profile, the ‘transcriptome’, of individual cells play a key role in brain aging, and could influence the progression of neurodegenerative diseases. Seung-Jae V. Lee and Seokjin Ham at the Korea Advanced Institute of Science and Technology, Daejeon, South Korea, reviewed current understanding of brain aging gained via transcriptomic profiling. Most aging brain regions display decreased synaptic function and plasticity, together with increased expression of immune response genes. Individual genotypes influence specific regional and cellular transcriptomic changes. The role of RNA-binding proteins and changes in expression of non-coding RNAs are of particular interest. The researchers call for widespread research in all ethnic groups, as most studies involve mainly Caucasian participants.

## Introduction

Aging is a nearly universal biological process associated with gradual deterioration in physiological and biochemical functions, including cognitive decline^1,2^. Aging is also a major risk factor for many neurodegenerative diseases, including Alzheimer’s disease and Parkinson’s disease^2,3^. This close relationship between aging and neurodegenerative diseases suggests the existence of common transcriptional and posttranscriptional gene regulation mechanisms in the brain.

Age-associated cognitive declines usually correlate with corresponding age-related structural changes in the brain, such as neuronal loss and synaptic changes^1,4^. However, the correlation is not universal. For example, changes in gene expression profiles in the brain during aging can precede apparent histopathological degeneration of the brain and appear to underlie age-associated cognitive declines at the molecular level^5–8^. Therefore, transcriptome analysis of brain aging offers a systematic molecular approach to understand the causes of neurocognitive aging and neurodegenerative diseases.

The transcriptome is defined as “the complete complement of mRNA molecules generated by a cell or population of cells”^9,10^. The information of cell populations is stored in the genome; therefore, transcriptome profiling is important for interpreting functional genomic elements^11^. The objectives of transcriptomics, or the study of the transcriptome, are to categorize all transcripts, to analyze the variants of expressed genes and to quantify the levels of transcripts under different conditions^12^. The subsequent functional characterization of the genes and transcripts of interest obtained from transcriptome analyses also helps validate their biological relevance.

Transcriptome profiling of brain samples from individuals of varying ages is a useful strategy for studying human brain aging by allowing the detection of overall age-associated transcriptome changes. Although transcriptome profiling alone generates descriptive information, assessment and interpretation of this information allows researchers to effectively design subsequent functional analyses of candidate genes and transcripts^11,13^.

Because human postmortem tissues are difficult to obtain, progress in transcriptome profiling of human brain aging has been very slow. However, international collaboration for the collection of human brain samples and subsequent generation of various sets of experimental data has paved the way for molecular biological research of human brain aging^14–16^. This collaboration has thus contributed to the comprehensive understanding of aging-related transcriptomic changes in the human brain.

In this article, we review and discuss studies based on transcriptome analyses of human brain aging (Table 1). We categorize the subtopics of transcriptome analysis into global gene expression changes, cell type-specific expression changes, gene coexpression networks, expression quantitative trait loci, and alternative splicing. Decreased synaptic function and increased immune function are prevalent in most analyses, in addition to other features that have been detected in some studies. Increased intersample heterogeneity is also an indispensable factor in the understanding of human brain aging. In addition, we address age-associated expression changes in various types of noncoding RNAs, including microRNAs (miRNAs), long noncoding RNAs (lncRNAs), and circular RNAs (circRNAs). Transcriptome analysis of human brain aging will pave the way for the early diagnosis and prevention of cognitive aging and age-associated brain diseases.

**Table 1:** Representative transcriptomic studies of human brain aging discussed in this manuscript.

Category (method)	Reference	Availability	Sample number	Brain Region	Individual number	Sex (M/F)	Ethnicity	Age (year)	Other information
mRNA (microarray)	Lu et al.^5^	Not available	30	1	30	18/12	Unknown	26−106	PMI
mRNA (microarray)	Erraji-Benchekroun et al.^21^	Not available	71	2	39	30/9	3 AA/1 As/28 C/7 H	13−79	COD, PMI, pH, drugs
mRNA (microarray)	Berchtold et al.^6^	GSE11882	177	4	58	29/29	9 AA/45 C/4 Unknown	20−99	PMI
mRNA (microarray)	Loerch et al.^7^	Not available	28	1	28	14/14	Unknown	24−94	PMI
mRNA (microarray)	Somel et al.^19^	GSE17757	23	1	23	18/5	Unknown	0−98	COD, PMI, RIN
mRNA (microarray)	Colantuoni et al.^18^	GSE30272	269	1	269	177/92	147 AA/4 As/112 C/6 H	0−80	PMI, pH, RIN
mRNA (microarray)	Kang et al.^17^	GSE25219	1,340	16	57	31/26	16 A/2 A/E/3 As/32 E/4 H	0−82	COD, PMI, pH, genomic abnormalities, facial or bodily abnormalities, gross brain abnormalities, histological evaluation, medical history, ethanol use, cigarette use, drug abuse
mRNA (microarray)	Mazin et al.^52^	SRP005169	12 (pooled)	2	30	19/11	6 AA/24 C	0−98	COD, PMI, RIN
mRNA (microarray)	Mazin et al.^52^	SRP005169	13	1	13	11/2	5 AA/8 C	0−98	COD, PMI, RIN
mRNA (microarray)	Soreq et al.^20^	GSE46706	1,231	10	134	99/35	134C	16−106	COD, PMI, pH, RIN
mRNA (microarray)	Soreq et al.^20^	GSE36192	607	2	305	204/101	305C	16−101	No provided
mRNA (microarray)	Somel et al.^19^	GSE18012	12	1	12	12/0	Unknown	0−98	COD, PMI, RIN
mRNA (microarray)	Beveridge et al.^68^	Not available	97	1	97	70/27	28 AA/66 C/1 H/2 Unknown	0−78	PMI, pH, RIN
lncRNA (RNA-seq)	Barry et al.^79^	Not available	11	1	11	8/3	11C	21−78	COD, PMI, pH, RIN

*A* African, *AA* African–American, *As* Asian, *C* Caucasian, *E* European, *H* Hispanic, *COD* cause of death, *PMI* postmortem interval, *RIN* RNA integrity number.

## Age-dependent transcriptome changes in the brain include decreased synaptic function and increased immune function

**Fig. 1:**
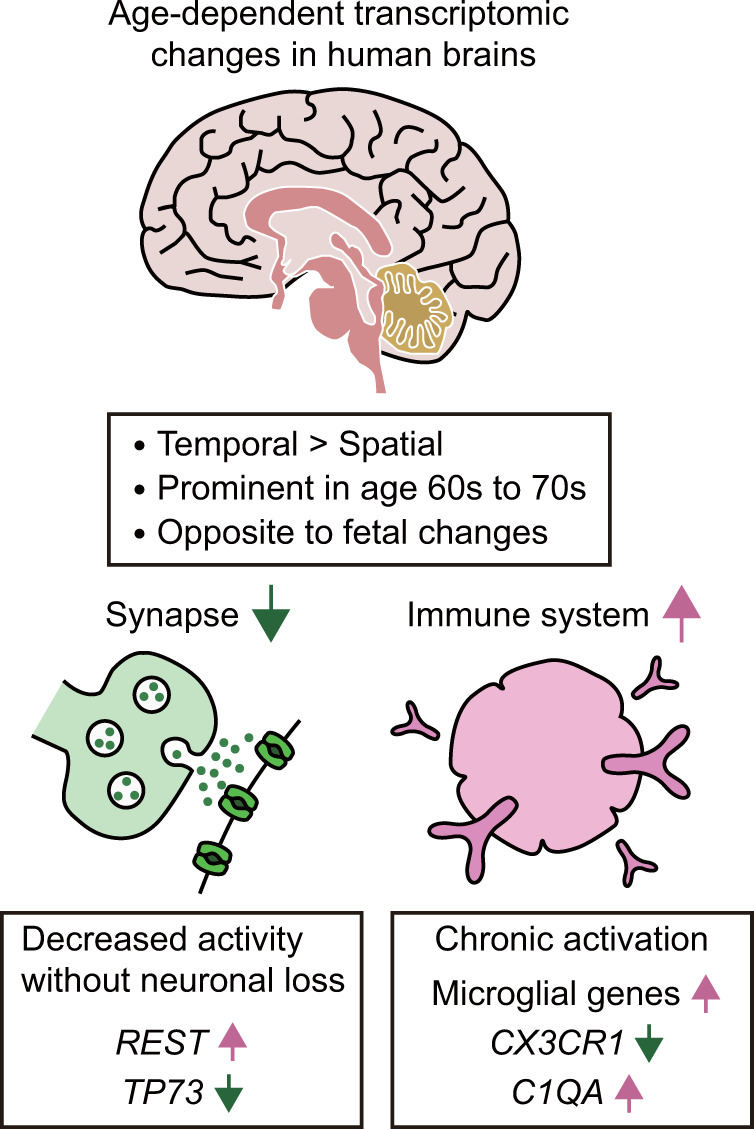
Global age-dependent transcriptomic changes in human brains. Expression changes are mainly temporal by relative preservation of their spatial identity. Substantial expression changes are detected in the sixth and seventh decades of human life. The directions of gene expression changes during adult aging tend to be opposite those occurring during fetal development. Throughout multiple brain regions, age-dependent transcriptomic changes include decreased synaptic function and increased immunity. It is worth noting that synaptic aging occurs even prior to neuronal loss. Decreased synaptic function underlies increased expression of repressor element-1-silencing transcription factor (*REST*) and decreased expression of the tumor protein 73 (*TP73*) gene. Increased immune responses are accompanied by upregulation of microglial genes and complement component 1q A (*C1QA*) and downregulation of genes encoding immunosuppressive factors, including C-X3-C motif chemokine receptor 1 (*CX3CR1*).

One of the most extensive transcriptome analyses on human brain aging with a large sample size and diverse ethnicity provided insights into global changes across the whole brain, as well as in specific regions^17^. Generally, temporal regulation of protein-coding genes is more pronounced than spatial or regional regulation (Fig. 1). The expression of 57.7% of genes is changed during prenatal development. In addition, 9.1% of the gene transcripts that are expressed in the neocortex are changed during postnatal development (an age of under 20 years), but only 0.7% of them are altered during adulthood (an age of over 20 years). These data indicate that the majority of differentially expressed genes display temporal changes during development and that the effects of aging on gene expression changes are relatively minor.

However, research using adult brains has indicated noticeable transcriptional changes during adult aging (Fig. 1). Another comprehensive transcriptome study analyzing the prefrontal cortex of 269 human individuals indicates that reduced rates of gene expression changes in the early postnatal brain are maintained throughout early childhood and through the first four decades of life^18^. Thereafter, substantial gene expression changes across multiple brain regions are detected in the sixth and seventh decades of human life, based on an analysis mostly including people of Caucasian ancestry^6,18^, indicating the existence of a qualitative transition phase (Fig. 1). The majority of the gene expression changes observed during adult aging are similar to those detected during postnatal development, which includes infancy, childhood, and puberty; interestingly, the directions of these changes are opposite those observed during fetal development^18,19^ (Fig. 1). In addition, males display a larger number of gene expression changes across multiple brain regions with aging than females^6,17^. In contrast to this sexual dimorphism, a study that analyzed two large data sets containing over 1800 human samples shows that the expression of genes exhibits similar changes with aging across brain regions^20^.

Notably, many independent transcriptome analyses have commonly indicated that genes that are upregulated during aging in most brain regions are enriched in inflammatory or immune-responsive genes annotated in the Gene Ontology (GO) database^5,6,20–25^ (Fig. 1). Although appropriate immune activation is neuroprotective, chronic immune activation appears to increase the vulnerability of the brain to neurocognitive aging and neurodegenerative diseases^22,23,26^. In contrast, multiple studies have repeatedly reported that genes that are downregulated across multiple brain regions during aging are enriched with genes related to synaptic transmission and plasticity^5–7,21,24,25,27,28^ (Fig. 1). These findings suggest that age-associated cognitive decline is driven by synaptic aging without neuronal loss^1,5^. Consistent with these observations, accumulating evidence indicates that dysregulation of intracellular calcium ion homeostasis is linked to age-related cognitive declines^29,30^. Genes that exhibit age-dependent expression changes in brain regions are enriched in other biological processes as well; these processes include neurotransmitter transport, energy metabolism, chaperone response, apoptosis, RNA metabolism, amino acid biosynthesis, DNA repair, mitochondrial function, and reactive oxygen species metabolism^5,19,21,27,31–33^. Many of these processes appear to be neuroprotective.

Recent transcriptomic studies in combination with thorough molecular genetic analysis indicate that repressor element-1-silencing transcription factor (REST), a neuronal repressor that is crucial for brain development, functions as a neuroprotective factor against brain aging^34^. The expression levels of *REST* are increased in normal aged brains but not in the brains of individuals with mild cognitive impairment or Alzheimer’s disease^25,34^. REST downregulates excitatory synaptic transmission and upregulates the longevity-associated transcription factor forkhead box O1 (FOXO1) in the mammalian brain, as well as its homolog DAF-16 in *Caenorhabditis elegans* to promote longevity^25^. These studies regarding REST suggest the existence of active protective mechanisms in the brain against neurodegeneration and the contribution of reduced excitatory synaptic transmission to longevity (Fig. 1).

Age-dependent gene expression changes in specific cell types have also been detected in diverse brain regions. Multiple papers have commonly reported that neuron-specific genes are predominantly downregulated in all brain regions during aging^7,19,21,33,35^, but these genes display region-specific, age-dependent expression changes by preserving their regional identity^20^. In contrast, glia-specific genes with age-dependent alterations in their expression lose their regional identity, and their expression is shifted to multiple regions^20^. Microglia act as innate immune cells of the central nervous system^36^. During aging, microglia-specific genes are generally upregulated in all brain regions, with the exception of the cerebellum^20,21,23,26^ (Fig. 1). In addition, endothelial cell-specific genes are transcriptionally upregulated across multiple brain regions during aging^20^. Oligodendrocytes act as supporting cells for neurons in the central nervous system by forming myelin sheaths around axons^37^. In contrast to the expression level changes in microglia-specific and endothelial cell-specific genes, the expression levels of oligodendrocyte-specific genes tend to decrease in various brain regions during aging^20,35^. These findings provide evidence supporting the occurrence of global and cell- or region-specific transcriptional changes during human brain aging.

## Gene coexpression network analysis supports decreased synaptic function and increased immune function during brain aging

**Fig. 2:**
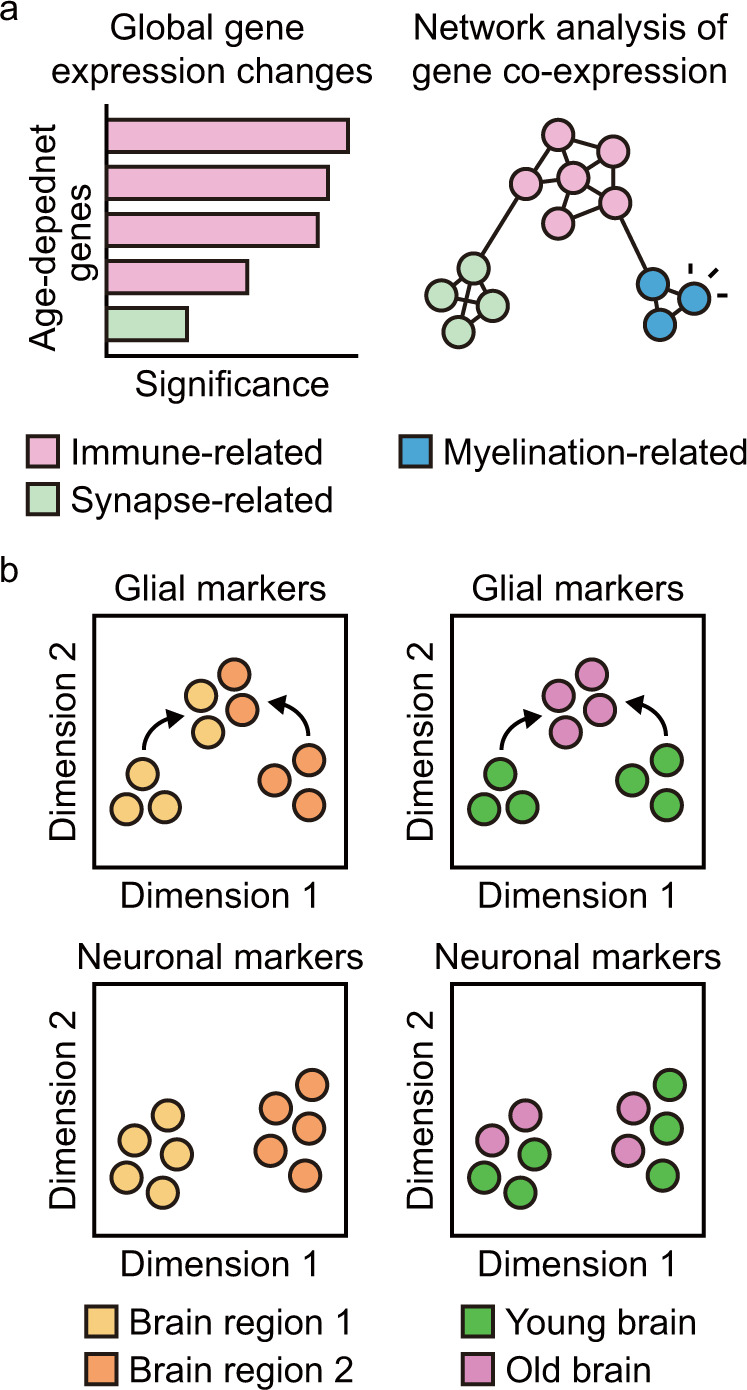
Targeted analysis of transcriptomic changes during human brain aging. **a** Age-dependent upregulation of myelination-related genes, which is undetectable in global analysis, is detected via gene coexpression network analysis. Genes that exhibit highly positive correlations among themselves are connected to one another. Different colors represent different groups of genes related to specific functions. **b** Glia-specific genes lose their regional identity during brain aging, whereas neuron-specific genes preserve their regional identity. Glial markers include genes that are specifically expressed in astrocytes and oligodendrocytes. The dimensions of the transcriptome are reduced for visualization by principal component analysis or multidimensional scaling.

Aging is regulated by complex interplay among various components;^2,3,8^ therefore, the transcriptomes of aged samples usually exhibit high intersample variation. Gene coexpression network analysis is utilized for detecting modules or clusters of highly correlated genes associated with specific functions and for obtaining key information embedded in heterogeneous transcriptomes^38^ (Fig. 2a). A network analysis of published transcriptomic data obtained from adult hippocampus samples has indicated that a module of genes that display age-dependent upregulation is enriched in myelination^39^. This study also confirmed an immune process-enriched module that correlated positively with age and a synaptic transmission-enriched module that correlated negatively with age^39^.

Although decreased synaptic function has been reproducibly reported in aged prefrontal cortices^40^, the increase in immune function in aged brains has been disputed by one study that analyzed three data sets obtained from the frontal cortices of 381 individuals^41^. Specifically, the expression levels of multiple genes in a microglia-specific module, including microglia surface receptors and Toll-like receptors, decrease in the prefrontal cortex with age^41^. Thus, some functions of microglia appear to decline during brain aging. However, the microglia module also contains genes encoding immunosuppressive factors, including C-X3-C motif chemokine receptor 1 (CX_3_CR1);^41^ therefore, the balance between immune activation and suppression is important for appropriate immune responses^6,22^. For instance, the microglia-specific neuroprotective receptor for fractalkine (FKN), CX_3_CR1, suppresses excessive immune responses via microglia activation in mice and rats^42,43^. Signaling through the FKN ligand and its receptor, CX_3_CR1, in microglia is reduced in aged brains^22,41,43^, and this reduction likely contributes to neurotoxic inflammation and results in neurodegeneration (Fig. 1). However, we cannot rule out the possibility that age-dependent downregulation of a subset of microglia-specific genes may reflect the complex regulation of these genes, as well as the altered number of microglia during aging. Collectively, these findings based on experimental data from multiple studies show that decreased synaptic function and increased immune function are predominant during human brain aging.

## Glia-specific genes lose their regional identity during brain aging

Examination of the age-dependent changes in the expression of cell-type-specific genes reveals that genes specific to glia, including astrocytes and oligodendrocytes, exhibit altered regional expression patterns during brain aging^20^ (Fig. 2b). These changes in the regional expression of glia-specific genes is most prominent in the hippocampus in the forebrain and in the substantia nigra in the midbrain, both of which are susceptible to Alzheimer’s disease and Parkinson’s disease pathogenesis. For example, the expression of astrocyte-specific genes in the hippocampus is similar to that observed in the cortex in young brains; however, the expression of these genes shifts toward that observed in intralobular white matter and the putamen in aged brains. Oligodendrocyte-specific genes exhibit differential expression in the hippocampus and in the substantia nigra in young brains; however, their expression in these structures becomes similar in aged brains. In contrast, neuron-specific genes preserve their regional identity during brain aging (Fig. 2b). Thus, during human brain aging, the expression of genes in specific cell types, including astrocytes and oligodendrocytes, changes dramatically and regional identities are lost.

## Analysis of expression quantitative trait loci (eQTLs) reveals that genetic backgrounds affect gene expression in an age-dependent manner

**Fig. 3:**
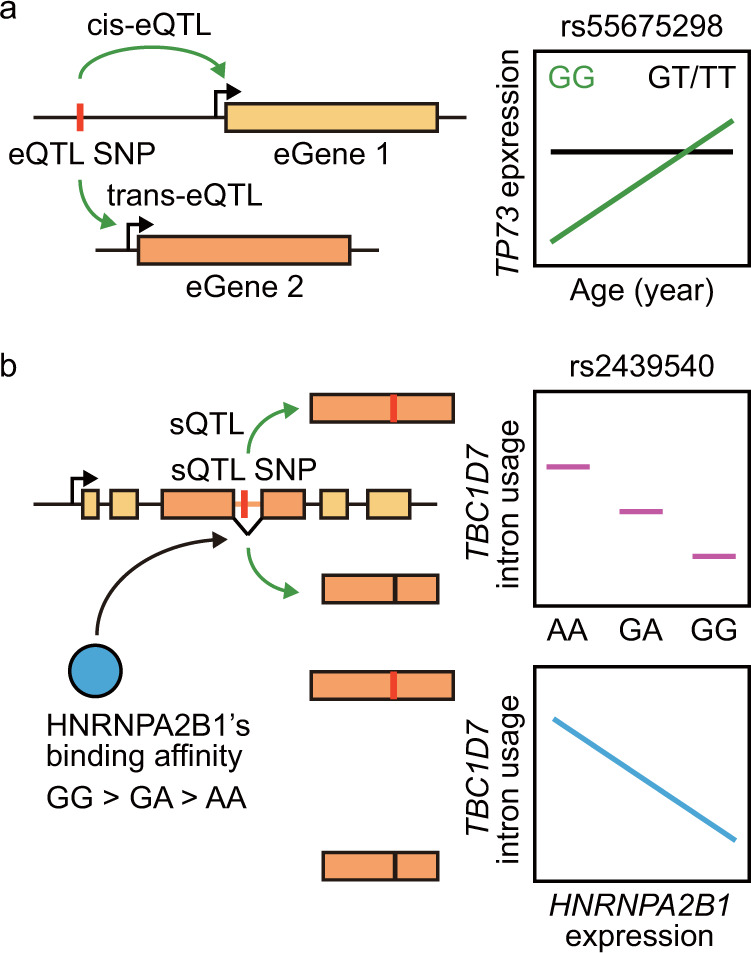
Genetic backgrounds that affect gene expression and alternative splicing in an age-dependent manner. **a**. An example of expression quantitative trait loci (eQTLs) during brain aging. Genotypes of eQTL single-nucleotide polymorphisms (SNPs) are associated with the expression of target genes (eGenes) on the same chromosome (cis-eQTLs) or on different chromosomes (trans-eQTLs). Individuals with the GG allele in the SNP rs55675298 exhibit an age-dependent increase in the expression of the tumor protein-coding p53 family gene *TP73*, whereas individuals with the GT or TT allele do not. **b**. An example of a splicing quantitative trait locus (sQTL) in aged brains. Genotypes of sQTL SNPs are associated with alternative splicing of target genes. The number of G alleles in the SNP rs2439540 correlates positively with hnRNP splicing factors, including HNRNPA2B1, and correlates negatively with the intron usage level of TBC1 domain family member 7 (*TBC1D7*). Consistent with these observations, the mRNA level of *HNRNPA2B1* correlates negatively with the intron usage level of *TBC1D7*.

Quantitative trait locus (QTL) analysis is a method that is used to infer the effects of genetic background on different traits. Expression QTLs (eQTLs) are genomic loci that are highly related to the expression levels of target genes^44,45^. Many eQTLs show consistent effects on the expression levels of their targets independent of age^17,18^. Interestingly, however, several studies using less stringent thresholds or a particular type of cell, such as microglia, have reported that some of these loci display differential effects on the brain at different ages^26,32^. One example is the single-nucleotide polymorphism (SNP) rs55675298^32^; individuals with the GG allele show an age-dependent increase in the expression of the tumor protein 73 (*TP73*) gene, which encodes a member of the p53 protein family, whereas individuals with the GT or TT alleles do not exhibit this increase (Fig. 3a). TP73 is critical for normal neuronal development and survival in mice^46–48^; therefore, age-dependent changes in the expression of *TP73* potentially affect brain aging and age-related diseases (Fig. 1). Indeed, the allele frequency of *TP73* is implicated in human Alzheimer’s disease^49^, and haploinsufficiency of *TP73* confers susceptibility to Alzheimer’s disease in model mice^50^. Similarly, the GG allele of the SNP rs72788737 is associated with age-dependent increases in the expression of complement component 1q A (*C1QA*), which encodes the C1q protein responsible for innate immune responses, whereas the GT or TT allele is not^32^. The expression levels of *C1QA* increase during normal brain aging (Fig. 1), and the C1q protein accumulates in several brain regions, including the hippocampus, substantia nigra, and piriform cortex, which are vulnerable to neurodegeneration^51^. In addition, the number of neuroprotective apolipoprotein E (*APOE*) gene ε2 haplotypes but not *APOE* ε4 haplotypes is associated with reduced expression levels of microglia-enriched genes in aged brains^26^. This pattern suggests that the *APOE* ε2 haplotypes moderate the excessive microglial activation that occurs during aging. Overall, these studies show the interaction between the genetic background and age-dependent gene expression changes, which may in turn affect human brain aging and age-associated diseases.

## Analysis of alternative splicing variants suggests the role of RNA-binding proteins in brain aging

In addition to gene expression changes, two notable papers that analyzed splicing changes have indicated that human brain aging is associated with alternative splicing^17,52^. Variations in alternative splicing are predominant during prenatal development^17^. After birth, 0.9% of the genes expressed in the neocortex exhibit differential splicing during postnatal development, whereas 1.4% of them do so during adulthood^17^. As the brain exhibits the highest level of splicing and preferential expression of genes encoding RNA-binding proteins among all organs of the body^16^, the 1.4% rate of age-dependent splicing changes is substantial compared with the 0.9% rate.

Remarkably, age-dependent splicing changes do not correlate with changes in the expression of the corresponding genes^52^. The splicing changes that occur during postnatal development and during subsequent aging processes display opposite trends;^52^ 75% of segments (exons or introns) show decreased inclusion in transcripts during postnatal development, whereas 77% of segments show increased inclusion during aging. The majority of segments that follow this “down–up” inclusion pattern are retained introns^52^. Transcripts with retained introns can be targets for nonsense-mediated decay (NMD), which is a crucial system for RNA quality control that degrades various mRNAs, including those with premature stop codons, and regulates alternative splicing^53^. Among the 15 of the 21 genes encoding NMD pathway components that are expressed in the brain, five [up-frameshift suppressor 3 homolog B (*UPF3b*), suppressor with morphological effect on genitalia homolog 1 (*SMG1*), *SMG5*, *SMG6*, and cancer susceptibility candidate 3 (*CASC3*)] follow the “down–up” pattern^52^. These findings suggest that components acting in the NMD pathway display characteristic age-dependent expression changes. NMD plays crucial roles in organismal longevity in invertebrates^54,55^. Thus, it will be interesting to evaluate whether the age-dependent expression changes in NMD pathway component-encoding genes contribute to brain aging or age-associated neurodegenerative diseases.

The expression levels of six genes encoding splicing factors, i.e., polypyrimidine tract-binding proteins 1 and 2 (*PTBP1* and *PTBP2*) and heterogeneous nuclear ribonucleoproteins A1, H1, H3, and F (*hnRNPA1*, *hnRNPH1*, *hnRNPH3*, and *hnRNPF*), follow the age-dependent “down–up” expression pattern^52^. The expression levels of these genes correlate positively with the “down–up” splicing pattern of particular genes and correlate negatively with the “up–up” or “up–down” splicing patterns of other genes. These relationships may reflect the idea that PTBP1 and PTBP2, which are located in the vicinity of spliced segments^56^, positively or negatively affect segment inclusion^57^. Indeed, a large proportion of PTB targets are segments that exhibit age-related splicing changes^35^.

Analysis of splicing QTLs (sQTLs), also called splicing-isoform QTLs (isoQTLs), identified 9,044 genomic loci associated with splicing events in 3,006 genes in the aged prefrontal cortex, including the SNP rs2439540^58^ (Fig. 3b). Although most of these sQTLs do not appear to affect gene expression, 42 loci correlate with the expression and splicing of the genes that contain the loci^58^. In addition, the 9,044 age-associated sQTLs are enriched with potential binding of 18 RNA-binding proteins (RBPs);^58^ PTBP1 is the most prevalently bound RBP, followed by HNRNPC, cleavage and polyadenylation specific factor 7 (CPSF7), and ELAVL1 [embryonic lethal, abnormal vision (ELAV)-like RNA-binding protein 1]. The age-associated sQTLs are also enriched with other hnRNPs^58^. For example, the frequency of the G allele of the SNP rs2439540, which correlates negatively with intron usage in the TBC1 domain family member 7 (*TBC1D7*) gene, correlates positively with binding of HNRNPA2B1 (Fig. 3b). Moreover, the mRNA levels of hnRNP splicing factors, including *HNRNPA2B1* and *HNRNPC*, correlate negatively with the intron usage levels of hundreds of genes (Fig. 3b), some of which are associated with Alzheimer’s disease^58^. These findings suggest that these RBPs, as well as PTBP1 and PTBP2, play roles in the age-dependent changes in splicing.

## The increased variability of gene expression indicates impairment of genome maintenance and repair with age

As described above, the transcriptome of the aged brain after the sixth and seventh decades of human life becomes similar to that of the fetal brain^18,19^. However, these changes are not consistently detected between cells or tissues, because the gene expression profiles of brains become heterogeneous during aging^5,27^. This increased variability of gene expression appears to be caused by accumulation of cellular damage and somatic mutations rather than by differences in germline mutations or aging rates between individuals^2,59–61^. Indeed, a pioneering paper that analyzed microarray data obtained from humans of various ages reported that DNA damage is highly enriched in the promoters of genes that are downregulated in aged brains^5^. This finding is consistent with reports showing that gene expression variability is more pronounced among age-dependently downregulated genes than age-dependently upregulated genes^33,60^. The expression of many age-dependently downregulated genes correlates with increased DNA damage^5^, indicating accelerated genome instability during brain aging. The susceptibility of specific brain regions, such as the hippocampus, substantia nigra, and basal ganglia, to DNA damage may be involved in the increased vulnerability of these brain regions to aging compared with that of other regions^62^. Prolonged accumulation of cellular defects often results in immune activation, which causes additional damage^63^, consistent with the increased immune responses observed in aged brains. In summary, age-dependent downregulation of genes associated with increased genome instability may underlie the accumulation of stochastic events in aged brain cells. Old cells subsequently become heterogeneous and inflammatory, and these changes may eventually lead to gradual declines in the physiological integrity of the brain.

## Noncoding RNAs that participate in human brain aging

**Fig. 4:**
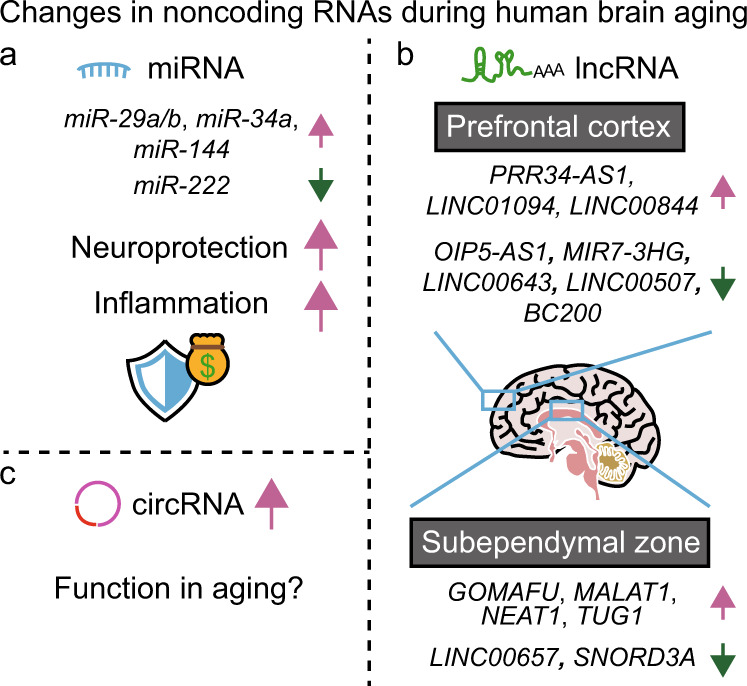
Noncoding RNAs that participate in human brain aging. **a** Several microRNAs (miRNAs) enhance neuroprotection in the elderly at the cost of inflammation. The expression of *miR-29a/b*, *miR-34a*, and *miR-144* increases with age, whereas that of *miR-222* decreases. **b** Long noncoding RNAs (lncRNAs) display age-dependent expression changes with preserved regional specificity. In the prefrontal cortex, the expression levels of the antisense RNA of the gene encoding proline-rich protein (*PRR34-AS1*), *LINC01094*, and *LINC00844* correlate positively with age. In contrast, the expression levels of the antisense RNA of the gene encoding opacity‐associated (Opa) interacting protein 1 (*OIP5-AS1*), MIR7-3 host gene (*MIR7-3HG*), *LINC00643*, *LINC00507*, and brain cytoplasmic 200 (*BC200*) correlate negatively with age. In the subependymal zone, *GOMAFU*, metastasis-associated lung adenocarcinoma transcript 1 (*MALAT1*), nuclear paraspeckle assembly transcript 1 (*NEAT1*), and taurine-upregulated gene 1 (*TUG1*) display age-dependent upregulation, whereas the expression of *LINC00657* and small nucleolar RNA, C/D box 3A (*SNORD3A*) is downregulated with age. **c** Circular RNAs (circRNAs) tend to accumulate during brain aging, but the function of their accumulation in aging remains unclear.

Transcriptomic studies have identified many novel classes of noncoding RNAs (ncRNAs) that are crucial for brain physiology^64,65^. Moreover, functional analyses have established ncRNAs as key regulators of brain aging^31,66,67^. In this section, we highlight the major classes of ncRNAs, i.e., microRNAs (miRNAs), long noncoding RNAs (lncRNAs), and circular RNAs (circRNAs), that participate in human brain aging (Fig. 4).

## Several miRNAs enhance adaptive neuroprotection in the elderly at the cost of microglial inflammation

Two independent studies that reported in-depth analysis of miRNAs indicated that expression changes in miRNAs are prominent during human brain aging^19,68^. Overall, the expression of miRNAs in human brains decreases with age after adolescence; in contrast, a subset of miRNAs display age-dependent upregulation^19,68^. In some cases, age-dependent expression changes in miRNAs appear to be protective against neurodegenerative diseases and brain aging. The expression of *miR-144* increases with age in the cerebellum and suppresses the expression of the ataxin 1 (*ATXN1*) gene^69^ (Fig. 4a). Because trinucleotide repeat expansion mutations in *ATXN1*, which augment the glutamine repeats in the encoded protein, underlie the development of spinocerebellar ataxia 1 (SCA1)^70^, miR-144 may play a protective role against this neurodegenerative disease. In addition, an age-dependent decrease in *miR-222* expression appears to result in derepression of its target genes^19^, including *REV1*/deoxycytidyl transferase, which regulates the DNA damage response^71^ (Fig. 4a). This process may eventually reduce the excessive DNA damage in aged brains.

However, the adaptive neuroprotection afforded by age-dependent expression changes in several miRNAs also seems to be associated with health complications, including excessive microglial inflammation^31^. For example, miR-34a, which acts as a tumor suppressor in neuroblastoma^72^, exhibits an age-dependent increase, which in turn downregulates neuronal genes, thus possibly participating in brain aging^19^ (Fig. 4a). In addition, the expression of *miR-29a/b* is dramatically increased with age in the superior frontal gyrus and the postcentral parietal area^19,73^ and exerts neuroprotective functions^74,75^, as well as induces excessive microglial inflammation^73^ (Fig. 4a). Low expression of *miR-29a/b* is observed in the brains of patients with sporadic Alzheimer’s disease and correlates with high BACE1/β-secretase levels^74^ and a high amyloid beta load^76^, again suggesting the neuroprotective role of miR-29a/b. Conversely, *miR-29a/b* expression correlates negatively with the mRNA levels of insulin-like growth factor 1 (*IGF1*) and *CX*_*3*_*CR1*^73^, both of which inhibit excessive microglial activation^42,43,77^. Thus, the age-dependent increases in miR-29a/b levels seem to protect against neurodegeneration in the elderly at the cost of excessive microglial inflammation (Fig. 4a).

## lncRNAs display high regional specificity in aging human brains

Multiple studies focusing on analysis of lncRNAs have reported that aged human brains exhibit expression changes in various lncRNAs with regional specificity^78–81^ (Fig. 4b). Among the 383 lncRNAs that are differentially expressed between young and aged samples from four brain regions, only eight exhibit consistent changes in all brain regions^80^, indicating the high regional specificity of lncRNAs.

In the prefrontal cortex, the expression levels of the antisense RNA of the gene encoding proline-rich protein (*PRR34-AS1*), *LINC01094*, and *LINC00844* correlate positively with age, whereas the expression levels of the antisense RNA of the gene encoding opacity‐associated (*Opa*) interacting protein 1 (*OIP5-AS1*), MIR7-3 host gene (*MIR7-3HG*), *LINC00643*, *LINC00507*, and brain cytoplasmic 200 (*BC200*) correlate negatively with age^78,81^ (Fig. 4b). These age-dependent changes in the expression of lncRNAs in the prefrontal cortex reflect cognitive declines with age. *LINC00507*, which has ribosome-binding activity and micropeptide-coding potential, is specific to cortical regions in primates^82^, suggesting that its downregulation with age is associated with cognitive decline. Another lncRNA, BC200, inhibits local mRNA translation in dendrites and synapses^83,84^. The expression level of *BC200* in the prefrontal cortex decreases by 65% between the ages of 49 and 86 years^78^ and correlates with age-dependent declines in synaptic functions^85^.

In the subependymal zone of the adult brain, which contains neural stem cells^86^, *GOMAFU* [also called myocardial infarction associated transcript (*MIAT*) and retinal noncoding RNA 2 (*RNCR*2)], metastasis-associated lung adenocarcinoma transcript 1 (*MALAT1*), nuclear paraspeckle assembly transcript 1 (*NEAT1*), and taurine-upregulated gene 1 (*TUG1*) exhibit age-dependent upregulation, whereas the expression of *LINC00657* and small nucleolar RNA, C/D box 3A (*SNORD3A*) is downregulated with age^79^ (Fig. 4b). GOMAFU acts as a scaffold for the sequestration of splicing factors and modulates the splicing of transcripts that are required for synaptic plasticity^87^. Neuronal activation decreases the expression of *GOMAFU* and releases splicing factors into the nucleoplasm^87^. In addition to GOMAFU, MALAT1 and NEAT1 are located in nuclear speckles and paraspeckles, respectively, where they regulate alternative splicing in neurons by sequestering splicing factors^88^. However, the mechanisms by which MALAT1 and NEAT1 participate in brain aging remain poorly understood. TUG1 sequesters miR-9, and its upregulation leads to release of BCL2 like 11 (*BCL2L11*) mRNA from miR-9 to promote apoptosis in rats with ischemia^89^. Thus, the age-dependent increase in the expression of *TUG1* in the subependymal zone may indicate the acceleration of neuronal apoptosis in aged brains^79^.

The age-dependent expression changes in a subset of lncRNAs also display specificity for other brain regions. The postcentral gyrus and superior frontal gyrus exhibit a larger number of differentially expressed lncRNAs than does the entorhinal cortex or hippocampus^80^. The majority of the age-dependent changes in lncRNA expression manifest as upregulation in all regions except the entorhinal cortex^80^. Overall, lncRNAs appear to be more region-specific than mRNAs in aging human brains.

## circRNA levels tend to increase during human brain aging

circRNAs are mostly noncoding RNAs that are formed by backsplicing events;^90^ however, the biological functions of circRNAs remain largely unexplored. Unlike linear RNAs, circRNAs are covalently linked at their termini and lack 3′ poly(A) tails; therefore, circRNAs display high resistance against exonucleases^91–93^. This property endows circRNAs with enhanced stability and raises the possibility that the levels of circRNAs remain high in nonproliferating cells. The levels of circRNAs do not correlate positively with those of their cognate mRNA isoforms^94,95^. circRNAs are highly expressed in brain tissues compared with nonbrain tissues in mice and humans^94^. Importantly, circRNAs display age-dependent accumulation in the brains of mice and a nonhuman primate, the rhesus macaque^95,96^ (Fig. 4c). In addition, certain circRNAs are specifically detected in patients with Alzheimer’s disease^97^. Therefore, although circRNAs have not been directly characterized in the aging human brain, it seems likely that the levels of circRNAs increase during human brain aging. It will be important to determine by future research whether the accumulation of circRNAs plays a role in aging and age-related diseases.

## Conclusions and perspectives

Transcriptome analysis of human brain aging offers a comprehensive view of neurocognitive aging and neurodegenerative diseases. Here, we reviewed studies regarding the transcriptome changes associated with human brain aging. Decreased synaptic function and increased immune function are prevalent in most brain regions during aging, but other age-associated transcriptome changes display cellular or regional specificities. Gene expression in glial cells, including astrocytes and oligodendrocytes, is dramatically altered during brain aging and is accompanied by loss of the regional identities of these genes. Age-dependent gene expression changes are also affected by genetic backgrounds and splicing events. The role of RBPs, including splicing factors, is important for the splicing events in many transcripts during brain aging. Increased intersample heterogeneity is a property that implies the impairment of genome maintenance and repair in aged brains. The role of age-associated expression changes in noncoding RNAs, including miRNAs, lncRNAs, and circRNAs, remains less well understood than those in mRNAs. Thus, future studies of these noncoding RNAs will lead to a deeper understanding of brain aging at the molecular level.

The emergence of single-cell RNA-seq technology has enhanced the potential of transcriptome analysis and will help improve the understanding of human brain aging at the single-cell level. Specifically, investigation of single-cell transcriptomes provides unbiased insights into the subtypes and the diversities of brain cells^98,99^. Single-cell RNA-seq overcomes the problem of bulk RNA-seq, i.e., that the transcriptional contribution from minor cell types is masked by major cell types in tissues. Currently, examination of single nuclei is usually preferred^98^. However, this method cannot estimate the expression of cytosolic transcripts. Therefore, optimization of single-cell isolation is required for brain transcriptome analysis at the single-cell level. A recent study reported successful analysis of the single-cell transcriptome in the aging mouse brain by introducing sophisticated brain tissue dissociation steps^100^. Thus, future research employing single-cell RNA-seq technology with human brain samples will pave the way for providing new insights into human brain aging. In addition, by combining this single-cell technique with in situ hybridization^101^, transcriptome analysis will allow spatial separation for visualizing gene expression in human brain cells during aging.

One of the limitations of previous research on transcriptome analysis of the human brain is that the majority of participants in postmortem brain studies are of Caucasian ethnicity. Among 1007 individuals with specified ethnicity in previous studies (Table 1), 733 (72.8%) and 32 (3.2%) were Caucasian and European, respectively. African-American (198, 19.7%) and African (16, 1.6%), Hispanic (18, 1.8%) and Asian (8, 0.8%) populations comprised a minor fraction. Thus, our understanding of human brain aging may be effective for only a subset of the human population at best. Future investigation of transcriptomes obtained from a wide spectrum of ethnic groups will be crucial for a comprehensive understanding of human brain aging.

In conclusion, understanding the transcriptome in aged human brains will lead to the early detection of cognitive aging and the development of interventions that delay brain aging at the molecular level. Furthermore, given the close relationship between brain aging and neurodegenerative diseases, the establishment of transcriptome-based hallmarks of human brain aging will facilitate the diagnosis and treatment of these disorders.
